# Engineering deceleration and acceleration of soliton emitted from Airy pulse with quadratic phase modulation in optical fibers without high-order effects

**DOI:** 10.1038/srep11843

**Published:** 2015-07-15

**Authors:** Lifu Zhang, Kun Liu, Haizhe Zhong, Jinggui Zhang, Jianqin Deng, Ying Li, Dianyuan Fan

**Affiliations:** 1 SZU-NUS Collaborative Innovation Center for Optoelectronic Science & Technology, Key Laboratory of Optoelectronic Devices and Systems of Ministry of Education and Guangdong Province, College of Optoelectronic Engineering, Shenzhen University, Shenzhen 518060, China; 2 School of Information Technology, Hunan First Normal College, Changsha 410205, China; 3 Advanced Fiber Resources (Zhuhai) Ltd., Zhuhai 519085, China

## Abstract

Soliton propagation direction can be engineered in optical fibers in the presence of high-order effects (HOEs). It is well known that Raman effects can decelerate the soliton. Here we investigate the manipulation of the deceleration or acceleration of soliton emitted from Airy pulse whose spectrum is imposed an initial quadratic phase modulation (QPM) in optical fibers in the absence of HOEs. We show that, under the action of the anomalous second-order dispersion (SOD) and Kerr nonlinearity, Airy pulse with QPM is able to emit soliton with acceleration or deceleration depending on whether the QPM is negative or positive, and at a rate that is determined by the magnitude of QPM. The reason is that the acceleration behaviors of incident Airy pulse is altered depending on whether SOD and QPM have the same or opposite signs. Our study shows the possibility of controlling and manipulating the soliton propagation and interaction in optical fibers without HOEs, by purposely choosing appropriate QPM parameter of an Airy pulse.

Airy wave packet is first found by Berry and Balazs as solution of the Schrödinger equation in the context of quantum mechanics^1^. It is impossible to realize practically because Airy wave packet carries infinite energy. As a result, it does not attract attention. Since truncated Airy beam with finite energy, which is also the solution of the Schrodinger equation^2^, was realized experimentally^3^ in 2007, it has drawn considerable attention^4^,^5^,^6^ because of its unique features such as quasi-non-diffraction, self-healing and transverse self-accelerattion^2^,^3^,^7^. These features make Airy beams useful for a variety of applications in optics, e.g., curved plasma channel generation in air^8^,^9^, light bullets generation^10^,^11^,^12^, all optical routing^13^, small particle manipulation^14^,^15^, high resolution microscopy^16^,^17^, and more.

Truncated Airy pulse, the counterparts of spatial truncated Airy beam, is capable of resisting dispersion and self-healing. In addition, Airy pulse propagates with their acceleration resulting from a change group velocity that manifests as self-acceleration or self-deceleration of the intensity peak of the pulse^18^. Truncated Airy pulse can be generated by launching the Gaussian pulse into a fiber with only third-order dispersion (TOD), and its acceleration or deceleration can be controlled by changing the sign of TOD^19^,^20^. It can also be produced by imparting a cubic spectral phase on an incident pulse through other pulse shaping techniques^21^. Extensive studies have been devoted to disclose the propagation dynamics of truncated Airy pulse from linear^10^,^11^,^18^,^22^,^23^,^24^,^25^,^26^ to nonlinear^27^,^28^,^29^,^30^,^31^,^32^,^33^,^34^,^35^ regimes. In linear optics, it was used for the realization of linear spatiotemporal light bullets^10^,^11^. The impact of the periodic dispersion modulation^22^, an initial frequency chirp^23^, and the second-order dispersion (SOD) as well as TOD^24^,^25^,^26^ on Airy pulse propagation has been reported. In the nonlinear regime, it is able to shed solitons under the effect of Kerr nonlinearity^27^. Zhang *et al.* investigated the modulation instability of Airy pulse^28^. Airy pulse can also be exploited to control supercontinumm generation^29^, self-focusing^30^, soliton self-frequency shift^31^,^32^, and soliton pair generation^33^.

In our previous work [23], we investigate the propagation dynamics of Airy pulse with an initial frequency chirp (FC). It was demonstrated that the joint action of the SOD and FC with the same sign leads to enhanced dispersion in the pulse shape; on the other hand, when the pulse dynamics is determined by SOD with a sign opposite to that of the FC, the Airy pulse first undergoes an initial compression, then reaches a breakup area, and then regenerates a new Airy pattern with an opposite acceleration. As FC is imposed on the incident Airy pulse, its temporal shape remains the same, while its spectral shape changes from Gaussian to Airy, and the direction of Airy tails is determined by the sign of the FC^23^. However, the situation changes oppositely when the spectrum of Airy pulse was imparted an additional *quadratic phase modulation* (QPM). Airy pulse with an initial QPM still keeps its spectral shape invariant, but its temporal shape will be distorted. In this paper, we are devoted to study the propagation dynamics of truncated Airy pulse with an initial QPM in linear and nonlinear regimes.

## Results

### Linear propagation of Airy pulse with an initial QPM

For linear propagation, the amplitude of Airy pulse 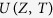 satisfies the following linear partial differential equation^36^:


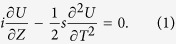


Equation (1) is readily solved by use of the Fourier-transform method. The general solution of Eq. (1) is give by


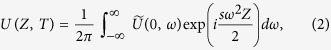


where 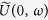 is the Fourier transform of the incident field at 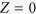 and is obtained by using


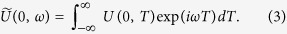


For Airy pulse with an initial QPM 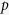, its spectrum expression is written as follow


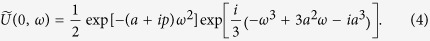


By inverse Fourier transform of Eq. (4), the expression of temporal shape is given by


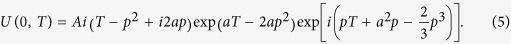


**Figure 1:**
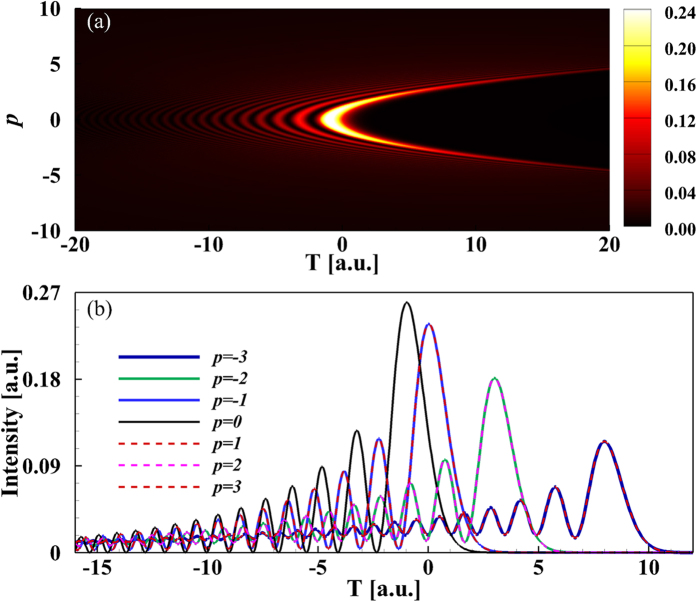
(**a**) Temporal evolution of Airy pulse as function of QPM parameter 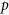. (**b**) Airy pulse shapes for some representative values of 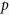.

Equation (5) shows the temporal shape of input Airy pulse with a QPM exhibits Airy distribution as well. Figure 1(a) depicts the temporal shape of such Airy pulse as a function of QPM parameter *p*. It is symmetric about the line 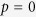, indicating the Airy pulse shapes are same for positive and negative QPM only if the absolute value of 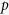 is equal. As the QPM was imposed on the incident Airy pulse, the Airy pattern changes as follows: first, its multipeak is delayed by an amount that increases with 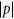, at the same time its peak intensity decreases; second, the oscillations of tails damp. The Airy pulse shapes are shown in Fig. 1(b) for some representative values of 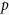. When 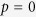, the oscillations are deep, with intensity dropping to zero between successive oscillation. For the case of 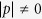, the peak of pulse is shifted toward right by the same amount only if the value of 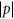 is equal, manifests through the overlapping of the shapes of Airy pulse with negative and positive QPM; and QPM induced peak intensity decrease is accompanied by the disappearance of oscillatory structure.

The spectral shape does not change because of linear propagation. Substituting Eq. (5) into Eq. (2), we obtain


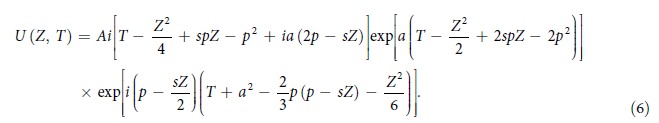


**Figure 2:**
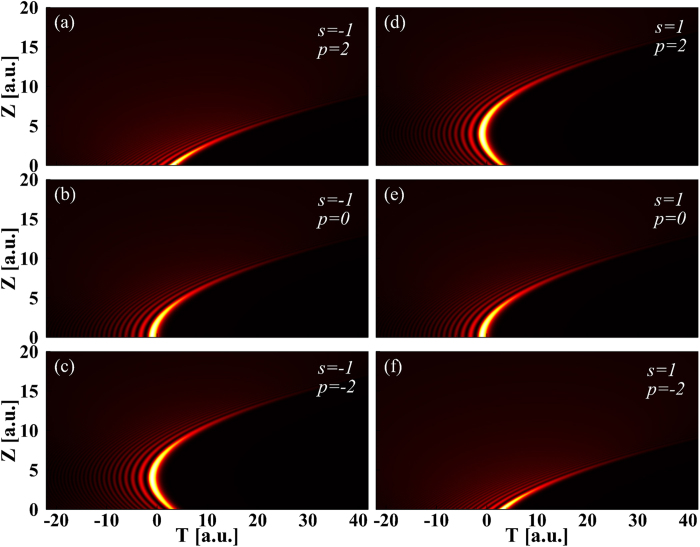
Temporal evolution of Airy pulse with different initial QPM (**a**,**d**) 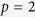, (**b**,**e**) 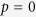 and (**c**,f) 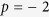 in the anomalous (left column) and normal (right column) dispersion regimes.

When 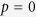 and 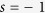, Eq. (6) covers the expression for spatial Airy beam^2^. According to the analytical expression of Eq. (6), Fig. 2 shows the temporal evolution of Airy pulse as a function of propagation distance for different values of QPM in the anomalous and normal dispersion regimes. When the QPM is not imposed on the incident Airy pulse, the propagation dynamics of Airy pulse is completely the same for the cases of anomalous (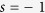, Fig. 2(b)) and normal (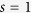, Fig. 2(e)) dispersions. However, the temporal evolution of Airy pulse changes considerably as an initial QPM was imparted on the input Airy pulse. In the normal dispersion regime (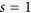), for the case of positive QPM 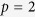 shown in Fig. 2(d), the Airy pulse first experiences an initial compression and displays acceleration, then reaches its maximum intensity, and then continues to propagate with deceleration. While for the case of negative QPM 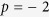 shown in Fig. 2(f), the Airy pulse is quickly distorted and always deceleration. A comparison of Figs. 2(a,d) (or Figs. 2(c,f)) shows the opposite occurs in the anomalous dispersion regime (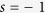). It can be found from Eq. (7) that as 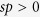, the value of 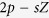 first decreases (or increase) from 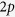 to zero and then increases with an increasing propagation distance 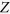 for the case of normal 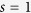 (or anomalous 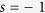) dispersion and positive (or negative) QPM. Therefore, the output Airy pulse at propagation distance 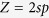, 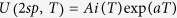, is equal to Airy pulse without QPM at 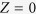, 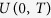. While for 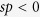, the value of 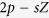 is always positive (negative) and increases (or decreases) from 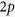 with an increase in the propagation distance for anomalous 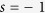 (or normal 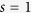) dispersion. Moreover, the dispersion effects is enhanced for 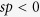.

**Figure 3:**
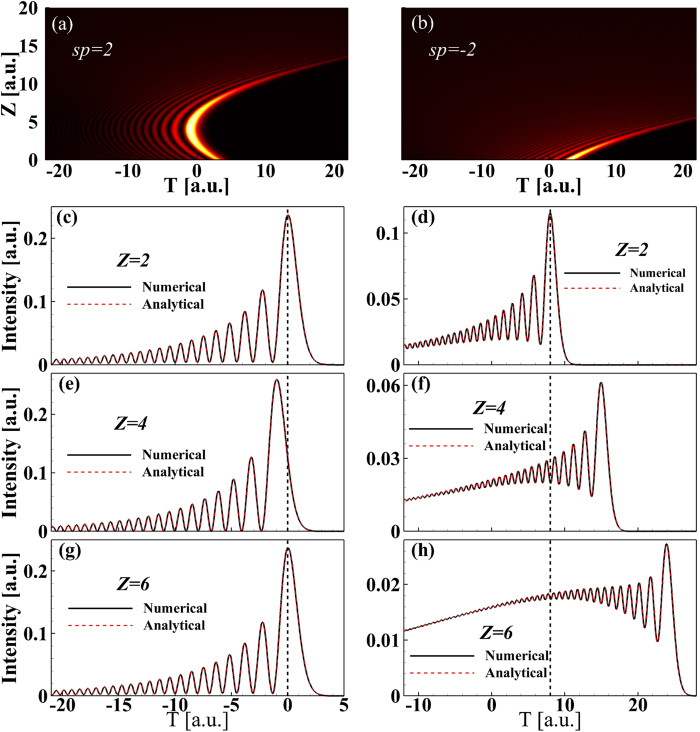
Temporal evolution of Airy pulse for the case of (**a**) 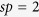 and (**b**) 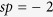. Comparison of pulse shape for Airy pulse between analytical (red dash lines) and numerical (black solid lines) results for (**c,e,g**) 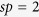 and (**d**,**f**,**h**) 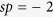 for several propagation distances.

To verify the analytical results shown in Figs. 1 and 2, we model Airy pulse with an initial QPM propagation with the Eq. (1) in an optical fiber by using the well-known split-step Fourier method^36^. Figures 3(a,b) show the temporal evolutions of Airy pulse for the cases of 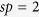 and 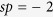, respectively. When 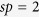, the propagation process of Airy pulse shown in Fig. 3(a) is the same as those obtained analytically shown in Figs. 2(c,d). Figure 3(b) corresponding to the case of 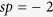 is the same as those obtained analytically shown in Figs. 2(a,f). This indicated that the numerical simulations confirm the novel propagation dynamics of Airy pulses with an initial QPM predicted by analytical analysis. In addition, Figs. 3(c–h) display a comparison of pulse shapes between analytical (red dash lines) and numerical (black solid lines) results at some representative propagation distances for 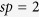 and 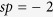. All red dash lines overlap with black solid lines. Once again, we get very good qualitative agreement between analytical results and numerical solutions of Eq. (1).

**Figure 4:**
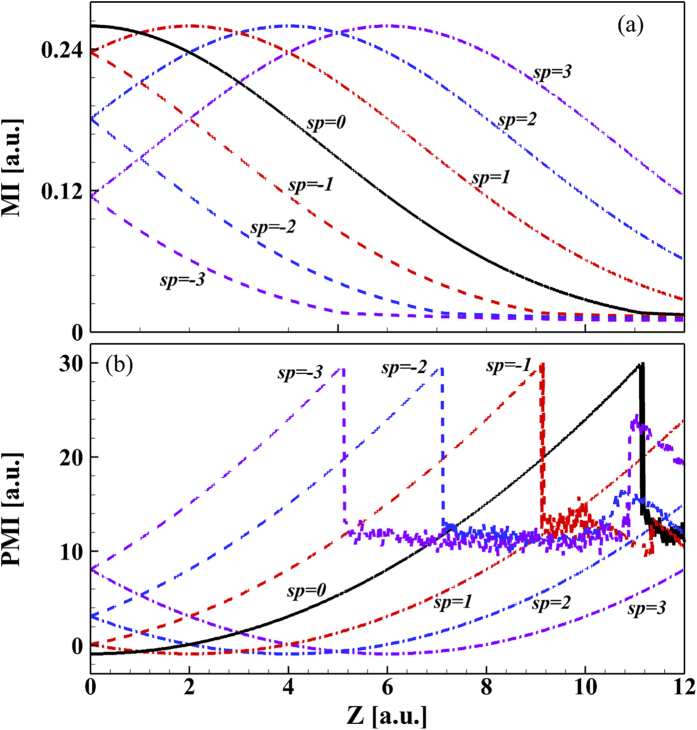
(**a**) Maximum intensity (MI) and (**b**) position of MI (PMI) are plotted as a function of propagation distance 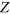 for several values of 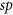 when Airy pulse with an initial QPM imposed propagates in the linear regime.

Figure 3(c) shows the Airy pulse shape at 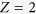; it should be compared with Fig. 3(g) where the pulse shape at 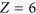 is shown. The pulse shapes are the same for both propagation distances. To get a better understanding of the impact of initial QPM on the Airy pulse linear propagation, Figs. 4(a,b) show the maximum intensity (MI) and the position of MI (PMI) as a function of propagation distances for several values of 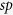. When 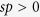, the curve of MI for 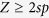 is a copy of the curve for 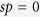; in the first 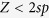 propagation distance, the MI increases and reaches maximum intensity. When 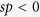, the curve can be obtained by shifting the curve of 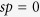 toward left by an amount of 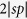. This is indicated that positive or negative QPM is able to advance or delay the Airy pattern breakdown during its propagation in linear media with normal or anomalous dispersion. It can be concluded from Fig. 4(b) in which the PMI is shown. This behavior can be understood in terms of the SPM-induced chirp as follows. When 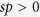, the dispersion-induced phase modulation adds to the initial QPM because the two contributions have the same sign. The situation changes for the case of 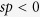. In this case, the contribution of the dispersion-induced phase modulation is of a kind opposite to that of initial QPM. As seen from Fig. 4 and Eq. (7), QPM becomes zero at a distance 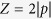. As a result, Airy pulse has no QPM (Fig. 3(e)).

### Nonlinear propagation of Airy pulse with an initial QPM

**Figure 5:**
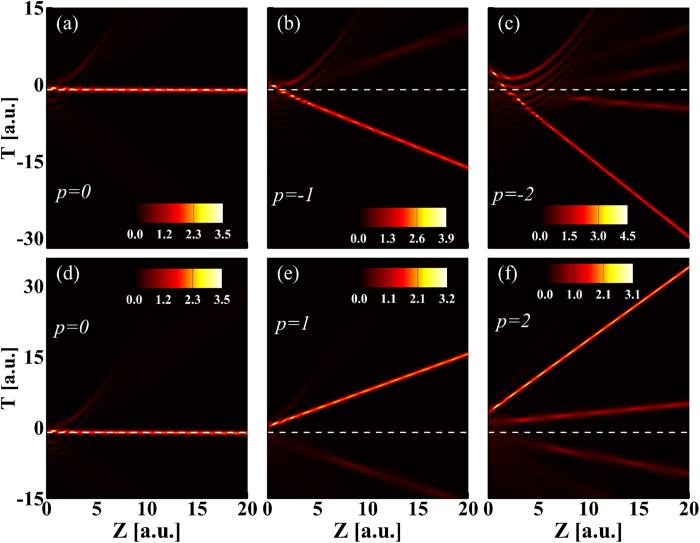
Temporal evolution of Airy pulse is plotted as a function of propagation distance for several values of QPM *p*.

Under the action of Kerr nonlinearity, the Airy pulse without QPM was distorted in the form of soliton shedding and dispersion background during propagation^27^. In the above discussion, we only consider the impact of QPM on the linear propagation of Airy pulse and find some new propagation behaviors. Do these unique linear properties make the nonlinear propagation of Airy pulse with QPM different from that of Airy pulse without QPM? Moreover, we move our attention on the nonlinear propagation of Airy pulse with an initial QPM. Figure 5 shows the temporal evolutions of Airy pulse with different values of QPM as a function of propagation distances in the anomalous dispersion regime. When 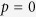, the soliton is shed from the main lobe of Airy pulse located in the vicinity of 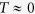 and propagates along a straight line (white dash line), indicating its velocity is not changed during propagation^27^,^34^. This is completely changed in the case of 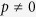. The main effect of QPM is to shift the shedding soliton peak linearly with propagation distance 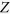. The shedding soliton is delayed or advanced depending on whether the sign of 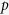 is minus or plus. When 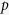 is positive, the QPM slows down the shedding soliton, and the soliton peak is delayed by an amount that increases linearly with distance. The opposite occurs as 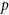 is negative. The initial QPM leads to shedding soliton with an enhanced rate of acceleration or deceleration that is determined by the sign and amplitude of 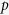. These results can also be applied for the case of spatial Airy beam with QPM. In addition, soliton shedding from Airy beams can also been manipulated at nonlinear interface by rotating the interface with an inclination angle^37^.

**Figure 6:**
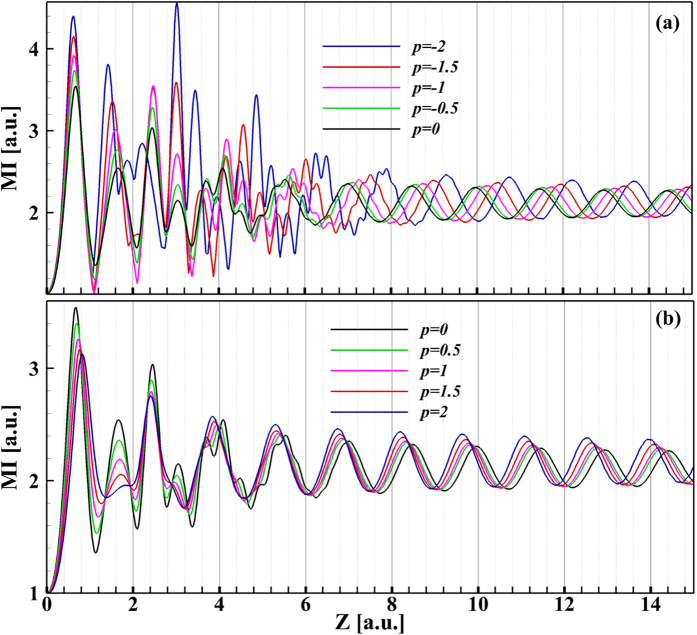
Maximum intensity is plotted as a function of propagation distance for several values of QPM *p*.

The MI of Airy pulse is plotted in Fig. 6 as a function of propagation distance for several values of QPM. Figure 6(a) shows the MI of Airy pulse with negative QPM. Under the combined contributions of anomalous GVD and self phase modulation (SPM), the main lobe of the Airy pulse undergoes an initial narrowing stage, leading to a quickly increasing of MI. As the MI reaches its maximum value, a soliton is formed out of the centered energy about the main lobe. The propagation distance required for shedding soliton decreases with an increasing 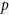. When 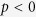, the shedding soliton experiences more successive collisions with side lobes because of its acceleration. As a result, the MI first exhibits a strenuous oscillatory structure and then reaches periodically stable evolution. However, for 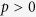, collisions between the shedding soliton with side lobes gradually decreases with increasing 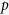 owing to the shedding soliton with deceleration. Moreover, the MI first exhibits a weak oscillation and then changes periodically. The soliton shed from Airy pulse with positive QPM achieves stability faster (after shorter propagation distance) than that emitted from Airy pulse with negative QPM, as the shedding soliton experiences less collisions with side lobes in the case of Airy pulse with positive QPM. The oscillations period is almost unchanged. It can also be seen from Figs. 5(c,f) that, for larger 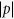, some weak solitons with acceleration or deceleration appear except for the intensive accelerating or decelerating soliton.

## Discussion

It is well known that the Raman effects or other high-order effects are able to decelerate the soliton. Can the soliton propagation direction be controlled in the absent of high-order effects? Our study indicates that the deceleration or acceleration of soliton can be manipulated for the nonlinear propagation of Airy pulse with QPM imposed by purposely choosing the magnitude and sign of its initial QPM. The Airy pulse imparted a QPM leads to novel linear and nonlinear dynamics. Although all analysis is performed in one dimensional media, all findings should hold in one and two dimensional spatial propagation cases in Kerr nonlinear media.

In summary, we have investigated the propagation dynamics of an Airy pulse with an initial QPM in an optical fiber by means of numerically simulation and analytically analysis. We obtain the expression for the linear propagation of Airy pulse with an initial QPM, and find that its propagation dynamics depends considerably on whether the QPM parameter 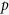 and fiber dispersion parameter 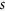 have the same or opposite signs. When 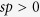, the Airy pulse with an initial QPM first experiences an initial compression, then reaches its maximum intensity, and then continues to propagate with the same acceleration. The Airy pulse with an initial QPM is always dispersed during propagation in the case of 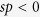. Under the combined effects of anomalous dispersion and Kerr nonlinearity, Airy pulse with an initial QPM is able to emit solitons with acceleration or deceleration depending on the sign of 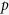, and at a rate determined by the magnitude of 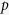.

## Methods

The propagation of Airy pulse in an optical fiber is carried out by numerically solving the well-known the nonlinear Schrödinger equation (NLSE) using the split-step Fourier method^36^. To simplify the model and broaden the applicability of the results, we normalize all the variables including the field that is normalized so that its peak input value is unity. The coordinates are normalized as follows: temporal coordinate 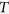 is normalized to the incident pulse width 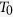, propagation distance 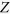 is measured in units of the dispersion length 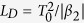, where 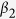 is the group velocity dispersion (GVD) parameter. The normalized NLSE then takes the form^36^


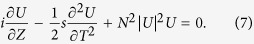


Here the parameter 
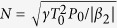
, represents the strength of the Kerr nonlinearity, where 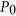 and 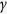 are the input peak power and the nonlinear coefficient respectively. 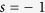 (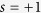) denotes anomalous (normal) GVD. It should be pointed out that, for Airy pulse with multi-peak structure, the width of the main lobe of Airy pulse 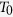 is usually used as a temporal scale.

## Additional Information

**How to cite this article**: Zhang, L. *et al.* Engineering deceleration and acceleration of soliton emitted from Airy pulse with quadratic phase modulation in optical fibers without high-order effects. *Sci. Rep.* **5**, 11843; doi: 10.1038/srep11843 (2015).
